# A multi-view feature representation for predicting drugs combination synergy based on ensemble and multi-task attention models

**DOI:** 10.1186/s13321-024-00903-3

**Published:** 2024-09-27

**Authors:** Samar Monem, Aboul Ella Hassanien, Alaa H. Abdel-Hamid

**Affiliations:** 1 Mathematics and Computer Science Department, Faculty of Science, Beni-Suef University, Beni-Suef, 62521 Egypt; 2 Faculty of Computer and AI, Cairo University, Cairo, Egypt

**Keywords:** Multi-task, Attention, Multi-view features, Copy number, Proteomics, Gene expression, Mutation, Drug target, Chemical drug features, Fingerprint, Multi-view graph, Synergy, Drug combination, Cancer cell line

## Abstract

This paper proposes a novel multi-view ensemble predictor model that is designed to address the challenge of determining synergistic drug combinations by predicting both the synergy score value values and synergy class label of drug combinations with cancer cell lines. The proposed methodology involves representing drug features through four distinct views: Simplified Molecular-Input Line-Entry System (SMILES) features, molecular graph features, fingerprint features, and drug-target features. On the other hand, cell line features are captured through four views: gene expression features, copy number features, mutation features, and proteomics features. To prevent overfitting of the model, two techniques are employed. First, each view feature of a drug is paired with each corresponding cell line view and input into a multi-task attention deep learning model. This multi-task model is trained to simultaneously predict both the synergy score value and synergy class label. This process results in sixteen input view features being fed into the multi-task model, producing sixteen prediction values. Subsequently, these prediction values are utilized as inputs for an ensemble model, which outputs the final prediction value. The ‘MVME’ model is assessed using the O’Neil dataset, which includes 38 distinct drugs combined across 39 distinct cancer cell lines to output 22,737 drug combination pairs. For the synergy score value, the proposed model scores a mean square error (MSE) of 206.57, a root mean square error (RMSE) of 14.30, and a Pearson score of 0.76. For the synergy class label, the model scores 0.90 for accuracy, 0.96 for precision, 0.57 for kappa, 0.96 for the area under the ROC curve (ROC-AUC), and 0.88 for the area under the precision-recall curve (PR-AUC).

## Scientific contribution

This paper presents an enhanced synergistic drug combination model by utilizing four different feature views for drugs and four views for cancer cell lines. Each view is then input into a multi-task deep learning model to predict both the synergy score and class label simultaneously. To address the challenge of managing diverse views and their corresponding prediction values while avoiding overfitting, an ensemble model is applied.

## Introduction

Addressing various biochemical processes within cells in complex diseases is limited by the effectiveness of a single drug targeting a single entity. Consequently, drug combination therapy, wherein multiple drugs are combined to produce improved therapeutic outcomes beyond the capabilities of individual pharmaceuticals, emerges as a viable strategy to overcome this limitation. The added advantage of reducing adverse effects by reducing the amount of drug needed for each treatment further enhances its appeal. Over several decades, the effectiveness of drug combination treatment has been demonstrated, specifically regarding the pervasive issue of drug resistance in cancer. So, identifying the best drug combination becomes a crucial endeavor with numerous implications for clinical, translational, and economic research. The efficacy of these drug combinations is significantly influenced by the synergy score value.

It is impractical to evaluate the synergy of drug combinations by experimental studies when dealing with a large number of drug combinations in high-throughput screens. Not only are such experiments perilous, but they are also costly, time-intensive, and demand considerable technical expertise, research experience, and human resources. So, deep learning models have become useful instruments in the field of biomedicine, offering more scalable and efficient simulation and analysis of biomedical data. Various approaches have been proposed to develop simulation models able to forecast effective drug combinations. These approaches leverage diverse factors, including cell line omics data as [1, 2], structural network interactions as [3, 4], and chemical drug properties as [5]. However, accurately determining synergistic drug combinations remains an ongoing research challenge, necessitating the development of more precise models.

This paper proposes a model not only enhances accuracy in predicting synergistic drug combinations compared to existing techniques but also holds significant potential for advancing drug discovery and development. By offering more reliable predictions, the model can streamline the early stages of drug screening, reduce the costs associated with experimental validation, and accelerate the identification of promising drug combinations. These improvements could lead to more efficient development pipelines and quicker delivery of effective treatments to patients, ultimately transforming the landscape of therapeutic innovation.

The proposes model is ‘MAEM’ a Multi-task Attention Ensemble Model designed for the concurrent prediction of drug combinations' synergy class label and synergy score value with an ensemble model to address the overfitting issue. Initially, multi-views of drug features are utilized, where the first view involves SMILES features extracted from the Mordred algorithm [6] using the RDKit tool [7]. The second view incorporates the drug's molecular graph, and a multi-view graph mechanism is employed to represent the drug's molecular graph. The third view represents a binary vector of drug-target interaction features, while the final view encompasses the drug's fingerprint.

Additionally, four views are integrated for cell line features, encompassing the gene expression profile of cell lines, copy number data, genetic mutation data, and proteomics data. To manage these extensive feature sets, two techniques are employed to prevent overfitting during model training. Firstly, each drug view paired with each cancer cell line is federated as input into the multi-task attention learning model. This model takes the two drug features intended for combination with the target cancer cell line and outputs both the synergy score value and synergy class label. This process results in sixteen views being input into the multi-task attention model, generating sixteen predictions for each synergy score value and synergy class label.

Finally, an ensemble model is applied, which takes the sixteen synergy score values and synergy class labels as separate inputs, aggregating them to produce the final prediction score. The proposed model demonstrates superior performance compared to other models in the comparative analysis.

The structure of this paper is set up as follows: “Related works” section delves into the related works applied in drug combination research. “The proposed model” section provides a detailed overview of MAEM, elucidating its main components and components. “Experimental results” section covers several topics, such as the model parameters, evaluation metrics, dataset used, and experimental outcomes. Lastly, the summary of MAEM is concluded in “Conclusion” section.

## Related works

Numerous techniques have been proposed for developing a learning model that is capable of forecasting synergistic drug combinations. First machine learning algorithms are learned to predict synergistic drug combinations such as Random Forest, Support Vector Machines ElasticNet, Gradient Boosting Machines, etc. Then, DeepDSC [8] pioneered the application of a deep learning model for forecasting synergistic drug combinations. Initially, the model utilizes the concatenated SMILES features of drugs and the gene expression data of the cell line as inputs for a deep learning model. This deep model comprises fully connected layers that learn the input features and produce the synergy as output. This approach demonstrates superior synergy outcomes, showcasing a notable 7.2% improvement over five well-established machine-learning techniques.

Following this, AuDNNsynergy [1] employs a deep learning model with omics data. This innovative approach posits that the cell line feature comprises three distinct components: gene expression, copy number, and mutation data. Three autoencoders are used to represent each component. Subsequently, the drug features are combined with the cell line features and provided as input to a deep learning model. The utilization of omics data with deep learning surpasses the performance of DeepDSC, which relies solely on a deep learning model.

The SynPred [9] model stands out from others by integrating various references including Zero interaction potency models, Bliss independence, highest single agent, and Loewe additivity. It utilizes comprehensive omics data for cancer cells, encompassing gene expression, copy number, methylation, global chromatin, metabolomics, microRNA, and proteomics data, along with chemical drug features. Employing an autoencoder for each cell line feature helps reduce the dimensionality of the omics data. Subsequently, these refined data are fed into a deep learning model for independent prediction of each synergy type.

Moreover, TranSynergy [10] introduces an alternative approach to representing drug features. It proposed a methodology based on gene–gene interaction and cell-line gene dependency features to simulate the cellular effects of drugs. Initially, it introduces a novel technique called Shapley Additive Gene Set Enrichment Analysis to identify genes enhancing the synergy of drug combinations. Subsequently, drug features are derived from 2041 selected genes identified through drug-target interaction, while cell line features are represented from the gene dependency or gene expression related to these 2041 genes. Lastly, these features are input into a deep learning model developed with a transformer architecture.

Also, DeepDDS [5], proposed a novel approach for representing drug features. Drugs are portrayed as a graph network, where atoms serve as nodes and chemical relations form the bonds of the graph. Subsequently, the drug graph input to a graph attention network to drug extract features. Simultaneously, the cell line is obtained from gene expression data, and a multi-layer perceptron is trained to extract cell features. The ultimate step involves concatenating the features of the two drugs and the cell line into a single vector and inputting it into fully connected layers to predict whether the drug exhibits synergy or antagonism.

PRODeepSyn [2] introduces a novel perspective on cell line features, focusing on the impact of protein–protein interactions and integrating this information with omics data from cancer cell lines. The model incorporates genomics and transcriptomics data, along with information on protein–protein relationships, into the ultimate cell line embeddings. For drug features, it combines the Morgan fingerprint with the SMILES descriptors. Lastly, a deep learning model with batch normalization is employed to output synergy score values, mitigating the model's reliance on initial parameters and enhancing generalization.

Subsequently, CGMS introduces an innovative deep-learning model that represents two drugs and a cell line as nodes within a complete graph and all nodes are interconnected. The drug nodes' features are expressed through drug fingerprints and RedKit descriptors. The cell line features are obtained from gene expression data with a variance feature selection technique, isolating the most informative 5000 genes. Subsequently, both drug and cell line features undergo processing through an autoencoder, resulting in an output of 256 features for each. The model then applies a heterogeneous graph attention network to extract features from the complete graph, and the final output vector from the graph is input into a multi-layer perceptron for simultaneous prediction of synergy and sensitivity scores.

Moreover, MultiSyn [4] introduces a multi-task synergistic model capable of concurrent prediction of both the synergy score value and synergy class label. This model employs a multi-view graph representation to extract molecular graphs for drugs and utilizes SMILES descriptors to represent chemical drug features. These features are concatenated to form the representation of drug features. For cell line features, the model utilizes gene expression while integrating the impact of drug-drug interactions in cancer cells through an attention mechanism. Finally, the features are concatenated and input into a multi-task model to output both the synergy score value and synergy class label concurrently.

Also, MTLSynergy [11] proposed a multi-task learning model aimed at predicting both the synergy and sensitivity scores of drug combinations. The model utilized fingerprint and molecular features for drug attributes, alongside cell expression for cell line features. Subsequently, an autoencoder was applied to each set of drug and cell line features to reduce dimensionality. These encoded features were then input into a deep neural network to concurrently generate synergy and sensitivity scores.

All previous works have represented drug features using one or two views, and the same approach has been applied to cell line features. Additionally, the interactions between drugs, between a drug and a cell line, and between genes within a cell line have a significant impact on accurately simulating the complex interactions that occur in the body, which is the fundamental idea behind drug combinations rather than administering each drug individually.

In this paper, the paper addresses the first issue by integrating multi-view representations for both drug and cell line features, allowing for a more comprehensive study and handling of their diverse properties. To manage the redundancy that can arise from multi-view representations, we propose an ensemble model. The second issue is tackled by incorporating a view that can simulate the interactions between two drugs, between genes within the cell line, and between the drug and these genes.

## The proposed model

The MAEM model introduced in this paper is designed to predict the synergistic effects of drug combinations. Initially, both drug and cell line features are fed into a multi-task attention deep learning model, generating both a synergy score value and a synergy class label indicating the nature of the drug combination (synergistic or antagonistic). Subsequently, an ensemble model is applied to aggregate the predictions. The first and second subsections delve into the discussion of multi-view features for drugs and cell lines, respectively. The third and fourth subsections cover the details of the multi-task attention and ensemble models, respectively.

### Drug multi-view features

To extract the drug features, four multi-view representations are applied.

The first view involves the extraction of chemical drug features by utilizing the SMILES representations retrieved from the public PubChem website. Then, the “Mordred” features [6] from DeepChem’s chemical informatics software [12] are used to convert these representations into descriptor feature vectors. Mordred is specifically designed to generate a wide range of molecular descriptors, which are quantitative representations of the chemical properties of molecules. By using Mordred, the molecular representations of drugs are transformed into descriptor feature vectors, resulting in an extensive array of numerical features. In this case, Mordred computes 1613 distinct numerical features for each drug, covering 43 different categories. These features capture various chemical properties, including topological, geometrical, electronic, and thermodynamic aspects of the molecules. By converting complex molecular structures into a structured, one-dimensional numerical form. Pre-processing steps include the removal of non-numerical features and attributes with zero variance leaving each drug with 394 informative features. To enhance consistency, the resulting features undergo normalization through the tanh-norm approach.

In the second view, graph molecular drugs are converted into graphs, in which every atom serves as a node, and the chemical bonds that connect them are edges. Then, four views of the graphs are extracted using a multi-view graph technique applied as in [4]. This technique can capture the graph embedding characteristics by applying four view representations with lowing time complexity make them to appropriate for multi-view cell line feature representation. The first view concentrates on labeling nodes within the graph, where each unique node in the graph is identified and assigned a distinct numerical value, which is then represented as a vector encoding these numeric values for each node. The second view utilizes the labels associated with each edge in the graph, considering all possible paths between nodes, including loops. The frequency of each path's occurrence is calculated, resulting in a vector that reflects the occurrence counts of all paths, as proposed in earlier research. The third view aims to extract the density of the neighborhood surrounding each atom by analyzing the shortest path length among them. The occurrence of each unique path length is recorded in a vector that captures the distribution of path lengths within the graph. Finally, the fourth view focuses on the labels of all possible paths between nodes, counting the frequency of each distinct label sequence along the paths. The resulting vector is constructed based on these label occurrences. To maintain consistency across views, each view vector is initialized with a fixed-length vector that is entirely composed of zeros. The output of each view is then used to modify this vector. The final step involves concatenating the four views that were acquired from the multi-view graph technique into a single vector, subsequently normalized using the tanh-norm approach.

The third view applies drug fingerprint features, capturing a concise and distinctive numerical depiction of a drug's chemical structure derived from molecular descriptors. This paper specifically employs the MACCS (Molecular ACCess System) fingerprint [13], which is a collection of structural keys utilized in cheminformatics and computational chemistry to represent molecular structures. Comprising 166 structural keys, each key corresponds to a specific structural pattern or fragment commonly observed in organic molecules.

The fourth and final view focuses on features associated with drug-target interactions. As the cell line is consists of genes and if the drug target cell line genes responsible for caner disease, the representation of drug according to its genes targeted is necessary. Initially, drug targets are gathered from the PubChem website for each drug. Subsequently, all drug targets are consolidated into a single vector. Finally, each drug is assigned a binary vector of the same size as the collected targets. A value of 1 is assigned if there is an interaction between the drug and a specific target and 0 otherwise. This methodology yields a vector of length 567.

### Cell line multi-view features

Cell line feature extraction can be categorized into four views. The initial view involves the utilization of gene expression features, encompassing the transcription of DNA into RNA (mRNA) and subsequently translated into functional proteins, reflecting the biological state and function of the cells. Gene expression data for this paper are sourced from the Cancer Cell Line Encyclopedia (CCLE), except the 'OCCUBM' cell, which is obtained from the Sanger Cell Model Passports (SCMP) project. The gene expression datasets are integrated by identifying the intersecting genes between them, yielding a feature vector of 19,067.

The second perspective centers on copy number variations, which represent alterations in the number of copies of specific DNA segments within a genome. This alteration can have profound effects on gene expression and are often associated with an individual's susceptibility to various diseases or conditions, including cancer. By analyzing these variations, we can gain insights into the genomic alterations that contribute to disease progression and drug response.

The third view introduces mutation features, which capture changes in the DNA sequence of specific genes. These mutations can lead to altered or dysfunctional proteins, potentially driving oncogenic processes or influencing how cells respond to treatment. The mutation feature vector is binary, where each gene is assigned a value of 1 if a mutation is present and 0 if no mutation is detected. This binary representation allows for a straightforward analysis of the mutational landscape across different cell lines.

Both the copy number variations and mutation data are sourced from CCLE, ensuring consistency in the genomic data used. The resulting feature vectors for copy number variations and mutations are of lengths 25,267 and 17,256, respectively, providing a comprehensive view of the genomic alterations present in the cell lines. These detailed genomic features are critical for understanding the molecular underpinnings of cancer and for developing targeted therapeutic strategies.

The fourth perspective delves into the comprehensive set of proteins generated by a cell under specific conditions, commonly referred to as proteomics data. This data offers valuable insights into the functional state of cells, as proteins are the primary executors of biological processes and are directly involved in most cellular activities. This perspective captures the dynamic nature of protein expression, reflecting how cells respond to various stimuli or environmental conditions. Initial cell line data are collected from the CCLE, and for cell lines absent in CCLE, information is obtained from the SCMP project. Similar to the approach in gene expression, the datasets are consolidated by identifying the proteins that intersect between them resulting in a set of 2411 proteins features.

It is noteworthy that the features of cell line views exhibit higher dimensionality compared to drug view features. To address the challenge posed by the elevated dimensionality of cell features, the variational autoencoder approach is employed. Each cell view serves as input to a distinct variational autoencoder [14], with the Kullback–Leibler (KL) loss defined in Eq. (1), producing a condensed cell view with a length of 256 that normalized using the tanh-norm method.

1 $$KL = \frac{1}{2} \sum^1 + \log \left( {\sigma^2 } \right) - \mu^2 - \sigma^2 ,$$

where *μ* and *σ* represent the mean and standard deviation of the distribution in the latent space, respectively.

### Multi-task attention learning model

**Fig. 1:**
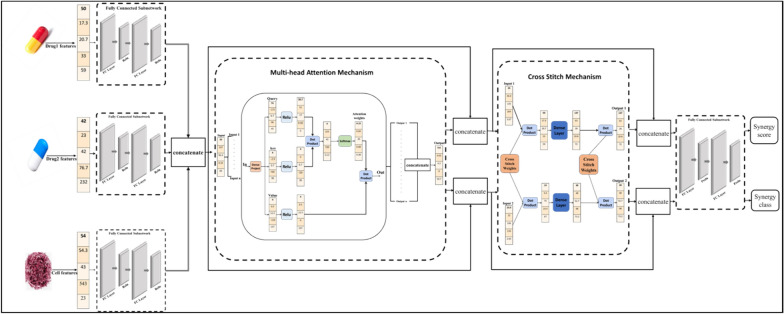
The structure of the multi-task attention model

This subsection is specifically dedicated to the simultaneous generation of both the synergy score value and synergy class label by integrating the feature views of both drugs and cell lines. Figure 1 shows the structure of the multi-task attention model.

Initially, the features of the two drugs are processed through separate fully connected subnetworks, each comprising three layers interconnected by the Rectified Linear Unit (ReLU) activation function, except the final layer which employs a linear activation function. To address potential overfitting, regularization techniques are applied to the weights and outputs of each fully connected layer. Concurrently, cell line features undergo a similar process in a distinct fully connected subnetwork to extract relevant information. Subsequently, the acquired features from both drugs and cell lines are concatenated and fed into a multi-head attention mechanism [15] to generate two distinct feature vectors representing the two target tasks.

The multi-head attention mechanism is a useful tool applied in multi-task models, utilizing attention weights to comprehend intricate relationships among feature vectors. It can accentuate informative vector values and disregard less significant ones by assigning trained weights to each vector value. This paper applied four heads for a multi-head attention approach, which enables the retrieved features to be weighted differently for each task.

The output of the multi-head attention is combined with the input using the concatenation process to collect a wider range of features and reduce the possibility of overfitting. These features are then fed into a cross-stitch mechanism [16] to learn relationships between tasks. By utilizing the cross-stitch subnetwork's capabilities, the model can discover and build relevant relations between tasks, facilitating knowledge transfer and improving prediction performance.

Thus, the relationship between the synergy score value and synergy class label tasks is learned, producing two output feature vectors for the two target tasks. Furthermore, for each task, the cross-stitch network's outputs and inputs are concatenated. Finally, the two resulting feature vectors are input into two separate fully connected subnetworks, each dedicated to one task. These subnetworks applied the PRELU activation function and output the synergy score value and synergy class label concurrently.

### Ensemble model

Each drug view is paired with each cell line view and fed into the described multi-task attention deep learning model, yielding sixteen input feature representations. The model outputs sixteen prediction values for synergy score values and class labels.

**Fig. 2:**
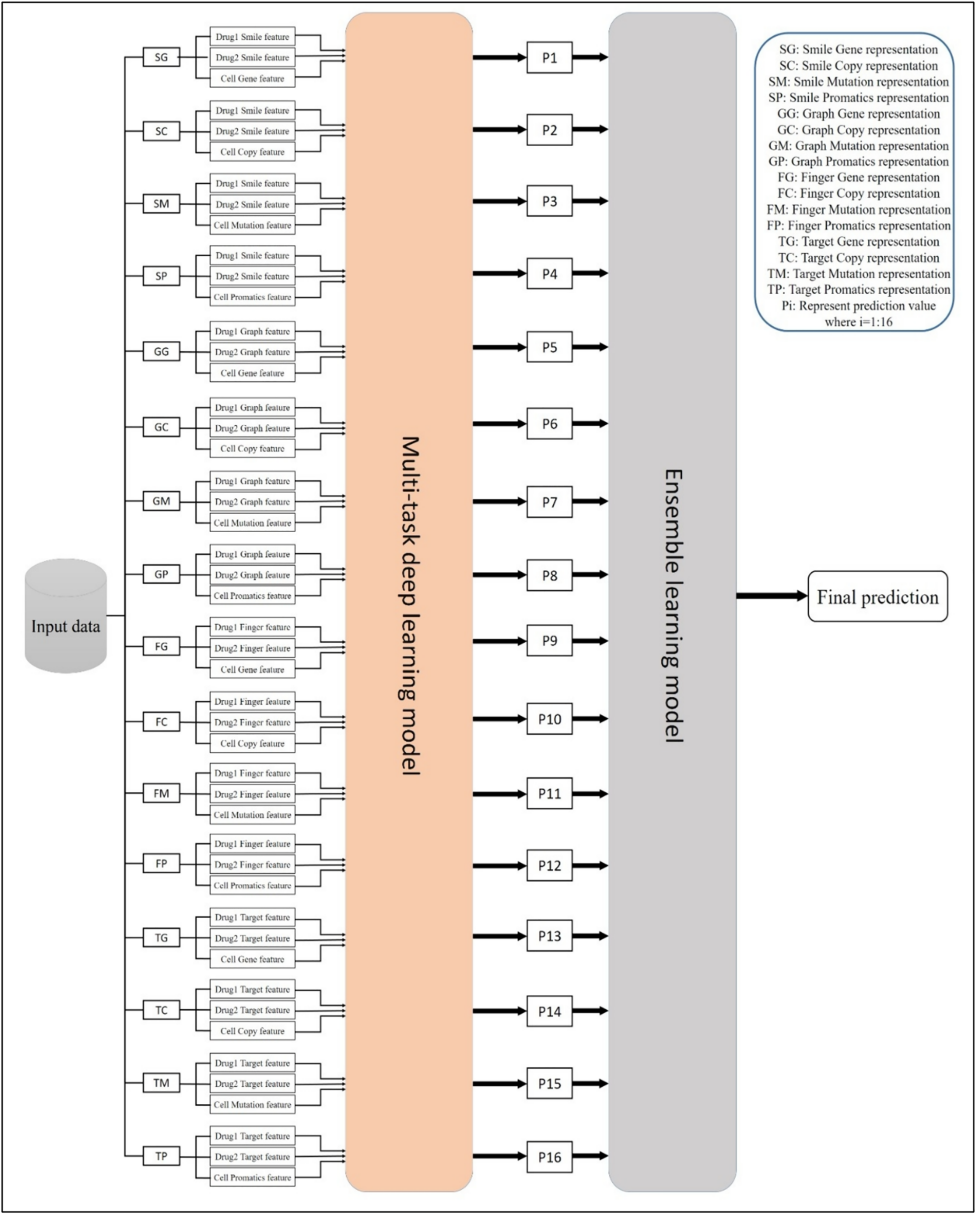
The structure of the MAEM ensemble model

To combine these predictions, an ensemble model is used, employing ‘input variation’ or ‘feature-level diversity’ by utilizing multiple inputs to train the same model structure as shown in Fig. 2. Training the same model structure on different subsets or transformations of the input features introduces diversity in the models' perspectives. This diversity can lead to a more robust and accurate ensemble when predictions are combined. The key idea is to create diversity among the models by varying the input data while keeping the model architecture consistent.

This paper applied the ensemble average model that typically involves simply averaging the predicted values from multiple models. This ensemble average can be a straightforward yet effective way to increase a model's generalization and robustness. It often works well when the individual models are diverse and complementary in their predictions. So, the sixteen prediction values are average to output the final prediction value for each synergy score value and synergy class label.

## Experimental results

This section assesses the performance of MAEM using a challenging dataset from O'Neil [17]. The first subsection presents dataset details, while the second discusses evaluation metrics. The third subsection covers model parameter settings, and the fourth subsection presents the experimental results of MAEM compared with other related works on the target dataset.

### Dataset characteristics

This paper utilizes a drug combination dataset obtained from the extensive O’Neil dataset, a widely acknowledged benchmark in cancer research. This dataset includes information on various drug combinations, specifying the names of paired drugs and the corresponding cancer cell lines designated for treatment. O'Neil dataset comprises 38 unique drugs tested across 39 diverse cancer cell lines, resulting in a comprehensive set of 22,737 drug combinations covering seven types of cancer tissues.

The Loewe Additivity score [18] is computed to determine the effect of a drug combination, whether it is antagonistic or synergistic. This score is based on a 4 × 4 dose–response matrix and supposes that no interaction between a drug and itself and ranges from − 326.46 to 179.12.

For the task of classification, the binary classification of drug synergy is applied. Specifically, drug combinations possessing a synergy score value exceeding 30 are categorized as synergistic, and those with a score value below 0 are deemed antagonistic. Combinations falling within the score range of 0–30 are not included in the training set, because they represent additive combinations, generating a balanced distribution of samples for the classification task. However, the removal of these samples in the synergistic classification task also entails eliminating the same samples in the regression task within this paper. To address this challenge, a three-class labeling strategy is implemented for the synergistic classification task. So, synergy score values falling below 0 belong to the antagonistic class, those exceeding 30 to the synergetic class, and core values between 0 and 30 to the additive class. This method produces an imbalanced distribution of samples among the three classes, potentially impacting classification training.

The dataset is divided into five cross-fold validations, ensuring that each drug-drug combination is included in only one-fold. In each cross-fold, one fold is used as the testing dataset, and the other four folds are used as the model's training dataset. The final results are then calculated throughout all five training runs and presented as the mean synergy score value and class label prediction scores.

### Evaluation metrics

Several regression metrics are used to assess the MAEM. The initial metric is the mean squared error (MSE), which measures the squared disparity between predicted and actual values. Also, the root mean squared error (RMSE) is calculated. In addition, the MSE’s 95% confidence interval is computed. Another crucial metric is the Pearson correlation coefficient (CC_P_) which assesses the degree of agreement between the predicted and actual values. To ensure the accuracy and dependability of results, the mean and standard deviation for each evaluation metric are proposed over the five folds, following the deployment of a five folds cross-validation approach.

On the other hand, for assessing the classification task for MAEM, various metrics are utilized to gauge performance. Initially, accuracy is employed to measure the model's ability to make correct predictions. However, given the imbalance in the test dataset as positive classes are rare, relying on accuracy metric may not offer a fair evaluation metric of the classifier's performance. Consequently, precision is utilized to assess the model's accuracy in predicting the synergetic class. In addition, Cohen's Kappa is used to determine how well the model performs by comparing its performance to that of a classifier that makes random guesses depending on class frequencies.

Furthermore, two crucial metrics are utilized which are especially useful for imbalanced classification tasks where a minority class has limited examples. These evaluation metrics are the receiver operating characteristic curve (ROC-AUC) and the area under the precision-recall curve (PR-AUC). The metric ROC-AUC assesses the classifier's capacity to differentiate between negative and positive samples under different threshold values, while the metric PR-AUC emphasizes the trade-off between precision and recall curve. This emphasis is particularly valuable when handling imbalanced data.

### Global model setting

To fully characterize the MAEM, various global parameters are outlined. Initially, the three hidden units for the fully connected subnetwork, managing two drug features and cell line features, are specified as [1024, 512, 256]. Moreover, the hidden units of the prediction subnetwork are defined as [1024, 128, 64] for both output tasks.

In the training phase, the model utilizes a learning rate set at 0.0001, processes batches of size 512, and undergoes 500 iterations. Optimization of the model is conducted through the AdamW optimizer [19], a modified version of the Adam optimizer that incorporates weight decay into the optimization learning. The model employs two key regularization techniques to mitigate the risk of overfitting, both of which are integral to enhancing the generalizability and predictive accuracy of the model on new data. First, weight decay, implemented through L1 and L2 regularization, is applied to all dense layers within the model. Specifically, an L1 regularization term of 0.001 and an L2 regularization term of 0.001 are used. This approach penalizes large weights, preventing the model from becoming overly complex and ensuring that it remains robust when exposed to unseen data.

In addition to regularization, the model undergoes a five-fold cross-validation process, as described in “Dataset characteristics” section on dataset characteristics. This method involves dividing the dataset into five subsets, where the model is trained on four subsets and test on the remaining one. This process is repeated five times, with each subset serving as the testing set once. Hyperparameters are optimized on one-fold are then applied consistently across the other four folds before training process. This cross-validation technique is crucial for ensuring that the model's performance is not reliant on a single partition of the data, thereby further reducing the likelihood of overfitting.

### Results and discussion

For comparing the MAEM model, four deep learning models (MutliSyn, PRODeepSyn, AudnnSynergy, DeepSynergy) designed for the drug combination task are chosen. These models are selected because they are applied to all samples in the O’Neil dataset. This selection is motivated by the challenge of integrating data from multiple sources, which may lead to slight variations in the values of collected data.

First, Table 1 outlines the performance metrics of different methods in predicting drug combination synergy score value. MAEM showcases superior performance with an MSE of 206.57, suggesting minimized prediction errors. Additionally, MAEM demonstrates the lowest RMSE values at 14.30. The Confidence Intervals signify the precision of estimates, with narrower intervals indicating more accurate predictions. Moreover, MAEM outshines other methods with a CC_P_ value of 0.78, underscoring its effectiveness in predicting drug combination synergy score value.

**Table 1:** Comparative analysis of synergy score value prediction performance with other related models

Method	MSE	Confidence interval	RMSE	CC_P_
MAEM	**206.57 ± 43.92**	**[152.04, 261.10]**	**14.30 ± 1.47**	**0.78 ± 0.03**
MTLSynergy	216.47 ± 37.32	[171.15, 265.79]	14.66 ± 1.26	0.76 ± 0.02
MutliSyn	219.14 ± 39.59	[170.00, 268.29]	14.75 ± 1.28	0.76 ± 0.02
PRODeepSyn	229.49 ± 42.81	[176.34, 282.64]	15.09 ± 1.37	0.75 ± 0.02
AudnnSynergy	241.12 ± 43.52	[187.09, 295.15]	15.46 ± 1.44	0.74 ± 0.03
DeepSynergy	255.49	[239.93, 271.06]	15.91 ± 1.56	0.73 ± 0.04

The best results are shown in bold

Table 2 shows how well the model performed in predicting the synergy class label when evaluating the classification of synergistic drug combinations in comparison to previous approaches that used the drug-drug combination pair approach. While the MAEM accuracy score isn't as good as other approaches, this can be related to the additive class, which is explained in “Dataset characteristics” section. It's crucial to remember that, as was already established, accuracy alone isn’t a fair and effective metric for unbalanced classification tasks. Nevertheless, MAEM outperforms all comparable models when evaluating additional metrics such as precision, ROC-AUC, and PR-AUC. MAEM continuously shows significant agreement, even though it might not have the greatest Kappa metric. As such, MAEM has exceptional performance in the categorization of synergistic drug combinations, especially when it comes to novel drug-drug combination pairs.

**Table 2:** Comparative analysis of synergy class label prediction performance with other related models

Method	Accuracy	PR-AUC	ROC-AUC	Precision	Kappa
MAEM	0.90 ± 0.02	**0.88 ± 0.02**	**0.96 ± 0.01**	**0.96 ± 0.02**	0.57 ± 0.05
MTLSynergy	**0.94 ± 0.01**	0.62 ± 0.05	0.90 ± 0.02	0.72 ± 0.06	0.51 ± 0.04
MutliSyn	0.90 ± 0.02	0.85 ± 0.03	0.95 ± 0.02	0.93 ± 0.01	**0.61 ± 0.06**
PRODeepSyn	0.93 ± 0.01	0.63 ± 0.05	0.90 ± 0.03	0.72 ± 0.06	0.51 ± 0.03
AudnnSynergy	0.93 ± 0.01	0.63 ± 0.06	0.91 ± 0.02	0.72 ± 0.06	0.51 ± 0.04
DeepSynergy	0.92 ± 0.03	0.59 ± 0.06	0.90 ± 0.03	0.56 ± 0.11	0.51 ± 0.04

The best results are shown in bold

**Fig. 3:**
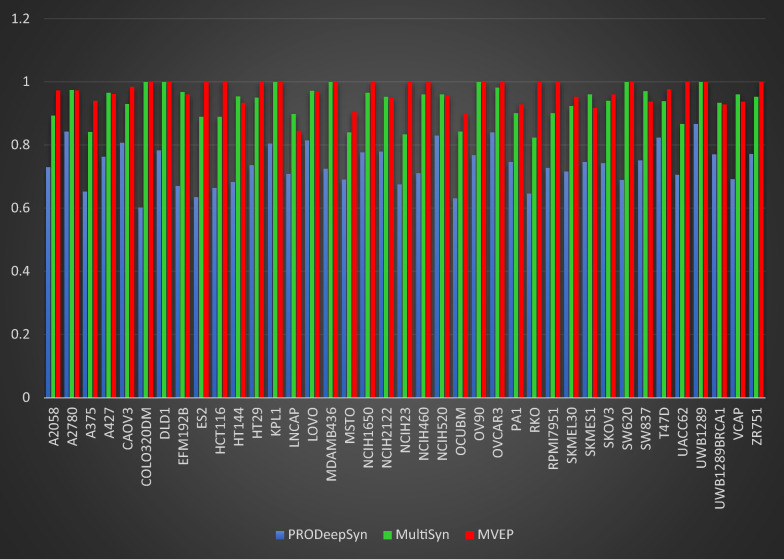
Comparative analysis of precision scores predictions across 39 cancer cell lines for MAEM, MutliSyn, and PRODeepSyn models

Furthermore, Fig. 3 illustrates precision values across various cancer cell lines, providing a comparative analysis between MAEM, MutliSyn, and PRODeepSyn. This visual representation aids in understanding the method's outcomes with alternative approaches. MAEM exhibits a significant improvement in precision scores compared to PRODeepSyn across all examined cancer cell lines. Additionally, it achieves competitive and enhanced precision scores relative to MutliSyn for the majority of the considered cancer cell lines.

**Fig. 4:**
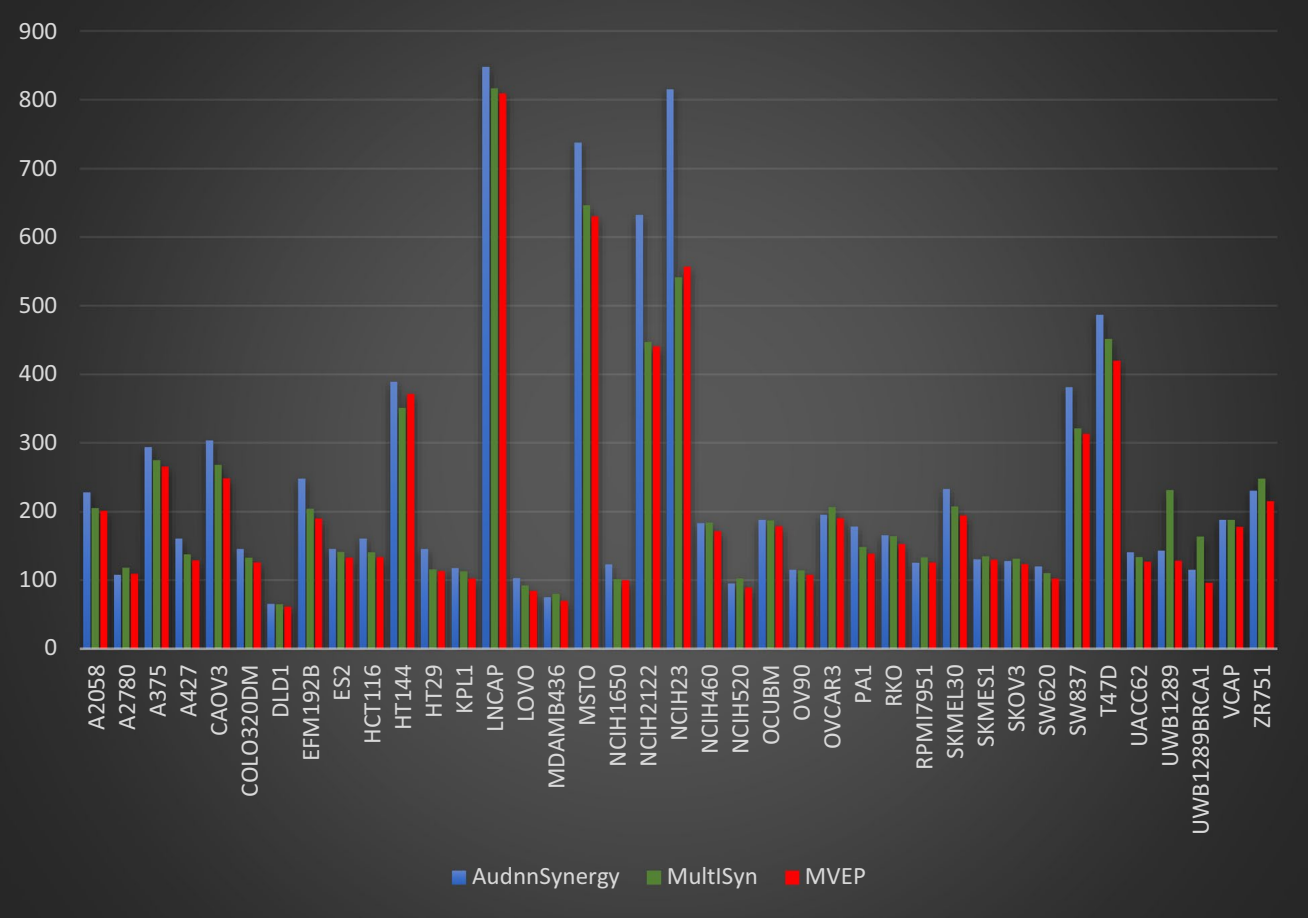
Comparative analysis of MSE predictions values across 39 cancer cell lines for MAEM, MutliSyn, and AudnnSynergy models

Moreover, Fig. 4 provides a thorough comparison of MSE scores across all cancer cell lines for MAEM, MutliSyn, and AudnnSynergy. MAEM consistently achieves competitive and improved MSE scores in contrast to both MutliSyn and AudnnSynergy across the majority of the assessed cancer cell lines.

To conduct a more in-depth analysis of the MAEM model and understand the individual significance of each feature in predicting regression synergy score values, Table 3 provides a breakdown of the impact of applying each feature independently in MAEM for classification metrics. The table initially reports the results for four cell line views featuring gene expression, copy number variation, mutation, and proteomics respectively. Following that, the results for four drug view features, encompassing target, mordred, graph, and fingerprint, are presented.

**Table 3:** Ablation study to assess the influence of individual drug and cell line view features in the MAEM model

Method	MSE	Confidence interval	RMSE	CC_P_
MAEM	**206.57 ± 43.92**	**[152.04, 261.10]**	**14.30 ± 1.47**	**0.78 ± 0.03**
Expression	208.23 ± 44.58	[152.89, 263.57]	14.35 ± 1.48	0.77 ± 0.03
Copy	208.39 ± 42.75	[155.31, 261.47]	14.36 ± 1.43	**0.78 ± 0.03**
Mutation	209.11 ± 42.74	[156.05, 262.17]	14.39 ± 1.42	0.77 ± 0.03
Proteomics	209.38 ± 45.77	[152.56, 266.21]	14.39 ± 1.52	0.77 ± 0.03
Target	209.96 ± 46.03	[152.82, 267.09]	14.41 ± 1.52	0.77 ± 0.03
Mordred	212.63 ± 46.62	[154.75, 270.52]	14.50 ± 1.54	0.77 ± 0.03
Graph	215.78 ± 42.80	[162.65, 268.91]	14.62 ± 1.41	0.74 ± 0.03
Finger	209.26 ± 40.25	[159.29, 259.23]	14.40 ± 1.35	0.77 ± 0.02

The best results are shown in bold

**Fig. 5:**
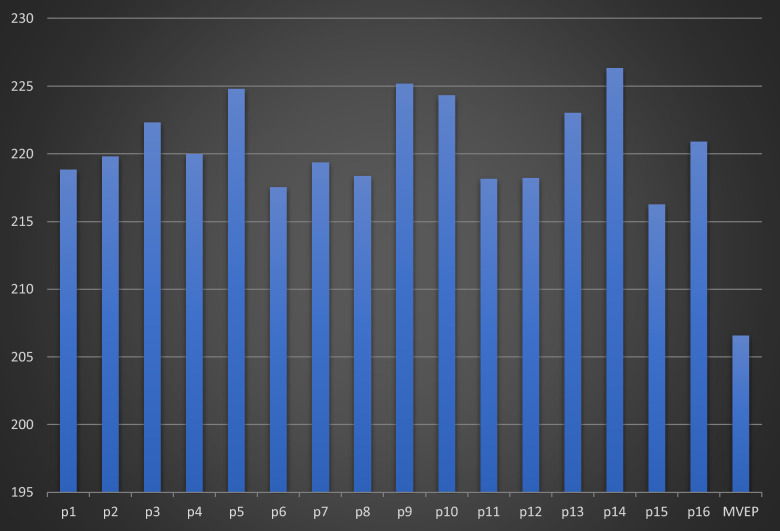
Comparative analysis of sixteen MSE predictions values output from sixteen representation input to multi-task attention model and MAEM

**Fig. 6:**
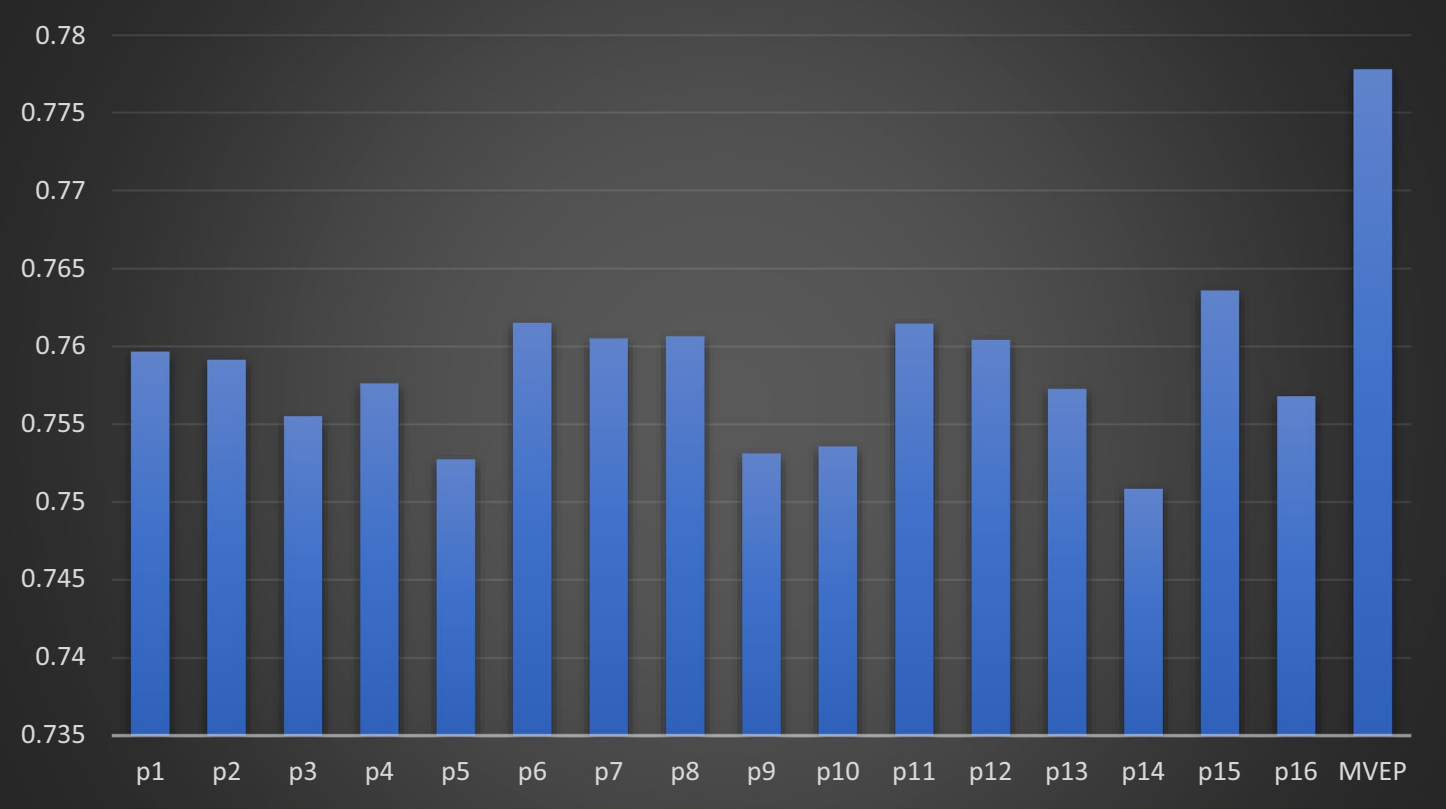
Comparative analysis of sixteen CC_P_ predictions values output from sixteen representation inputs to multi-task attention model and MAEM

Moreover, to analyze the role of ensemble learning in predicting both synergy score value and synergy class label, Fig. 5 illustrates the sixteen MSE prediction scores for each representation input to the multi-task attention model. Notably, MAEM consistently demonstrates low MSE across all sixteen models, indicating its significant efficacy. Here, p_i_ (i = 1:16) represents the output prediction of the sixteen models. Additionally, Fig. 6 showcases the CC_P_ between the sixteen models and MAEM. The results indicate that MAEM exhibits an improved CC_P_, surpassing the best prediction score among the sixteen models by nearly two points.

**Fig. 7:**
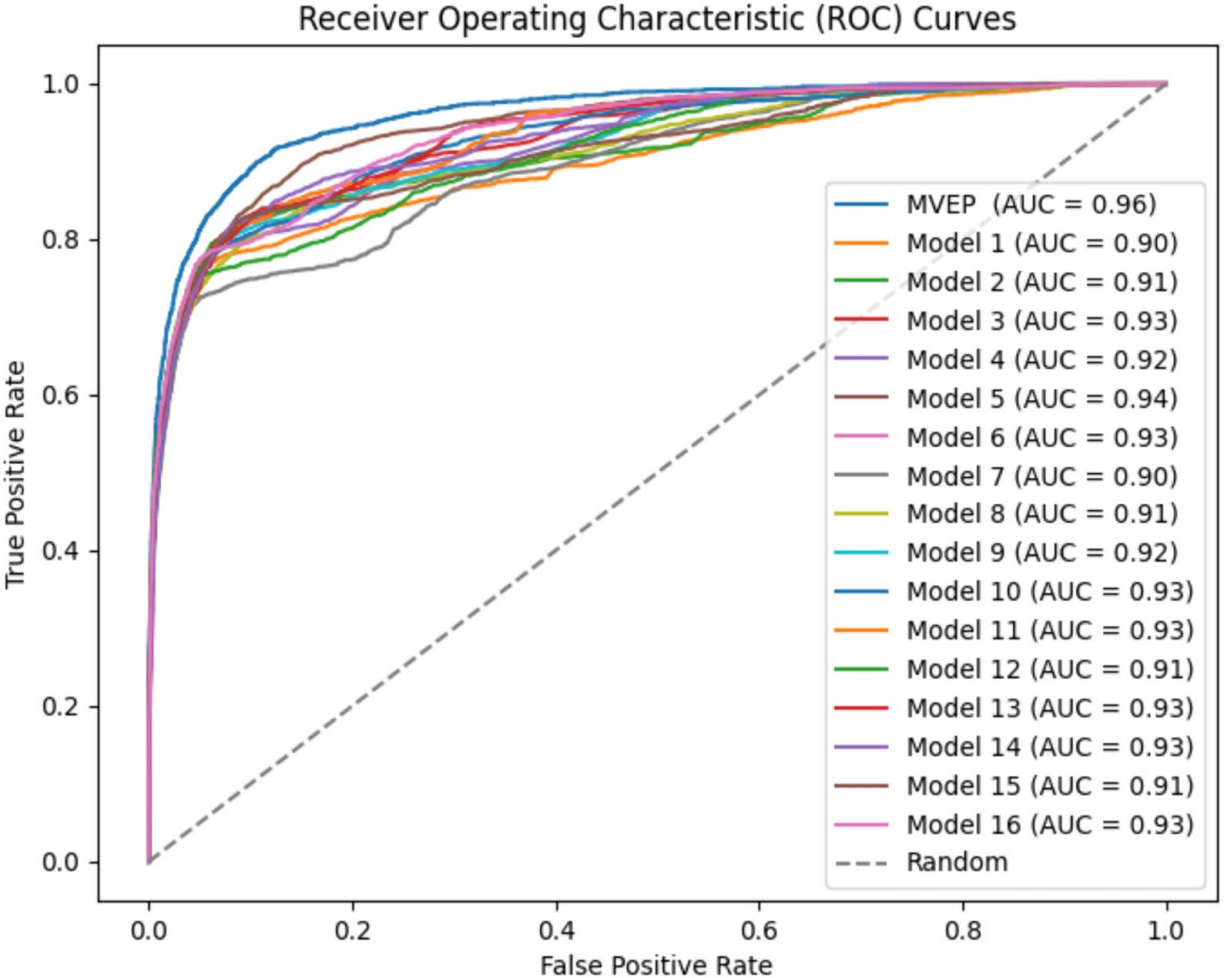
Comparative analysis of sixteen ROC-AUC prediction values output from sixteen representation inputs to multi-task attention model and MAEM

**Fig. 8:**
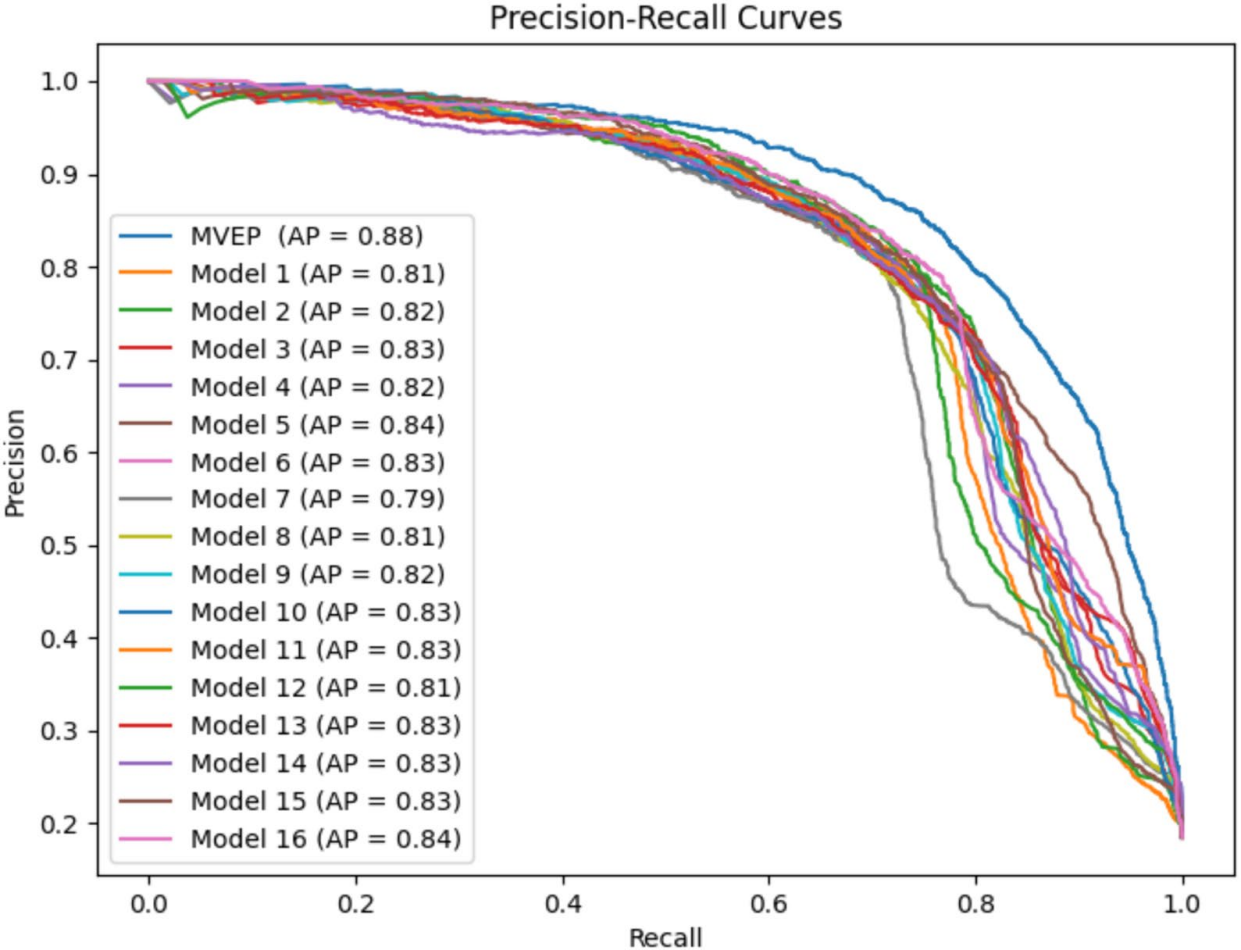
Comparative analysis of sixteen PR-AUC predictions values output from sixteen representation input to multi-task attention model and MAEM

For the classification task, highlighting the impact of the ensemble model, Figs. 7 and 8 depict the ROC-AUC curve and PR-AUC curve between the sixteen models and MAEM, respectively. These visuals reveal that MAEM enhances both the ROC-AUC and PR-AUC curves compared to the sixteen individual predictions. The ROC-AUC sees a 3% improvement over the best prediction among the sixteen models, while the PR-AUC experiences a 4% enhancement over the best of the sixteen models.

Tables 4 and 5 explore the effectiveness of different approaches to handling feature redundancy and learning tasks in drug synergy prediction.

**Table 4:** Ablation study to evaluate the impact of feature redundancy in drug and cell line views, as well as the effect of single-task learning on the prediction of synergy scores

Method	MSE	Confidence interval	RMSE	CC_P_
Concat	217.37 ± 47.42	[158.49, 276.24]	14.66 ± 1.54	0.76 ± 0.03
Single_task	216.38 ± 43.41	[162.49, 270.27]	14.64 ± 1.41	0.76 ± 0.03

**Table 5:** Ablation study to evaluate the impact of feature redundancy in drug and cell line views, as well as the effect of single-task learning on the prediction of synergy class label

Method	Accuracy	PR-AUC	ROC-AUC	Precision	Kappa
Concat	0.89 ± 0.02	0.83 ± 0.03	0.93 ± 0.02	0.93 ± 0.03	0.54 ± 0.03
Single_task	0.85 ± 0.03	0.68 ± 0.06	0.87 ± 0.02	0.90 ± 0.07	0.31 ± 0.09

In the Concat method, features from four drug views and four cell line views are concatenated and fed into a multi-task attention model. This approach shows how an ensemble model can address the redundancy problem by integrating multiple feature sets into a single representation, which is then used to predict the synergy score or class label.

The Single_task method, on the other hand, treats each drug and cell line view separately, applying them to a deep learning model with three fully connected layers. The resulting features are concatenated and passed through a fully connected subnetwork to predict either the synergy score or class label. The final output is derived by applying an ensemble model to the sixteen predictions generated by this process. This method highlights the differences between multi-task and single-task learning, emphasizing how single-task learning involves separate predictions for each view before combining them.

As shown, MAEM outperforms Concat and Single_task methods highlighting its superiority in managing redundancy and multi-task learning.

**Fig. 9:**
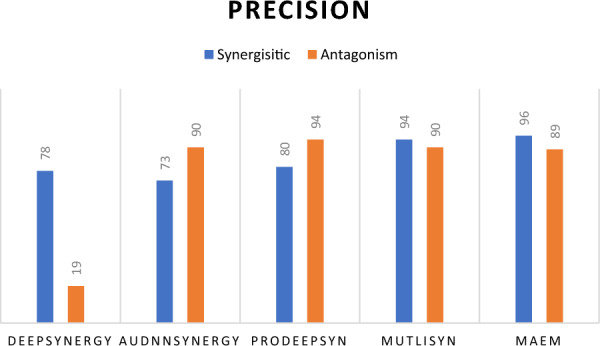
Comparative analysis of sixteen PR-AUC predictions values output from sixteen representation input to multi-task attention model and MAEM

Figure 9 presents the precision for both synergistic and antagonistic classes. The MAEM model achieves the highest precision for the synergistic class at 96%, demonstrating strong performance. For the antagonistic class, it has a precision of 89%, which is slightly lower than other models, indicating a marginally reduced accuracy in this category.

**Fig. 10:**
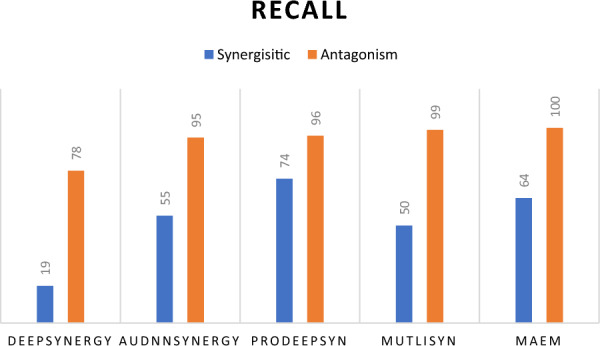
Comparative analysis of sixteen PR-AUC predictions values output from sixteen representation input to multi-task attention model and MAEM

Figure 10 illustrates the recall for the synergistic and antagonistic classes. MAEM excels in identifying antagonistic cases with a perfect recall of 100%. However, while it performs well for the synergistic cases, it does not surpass Prodpred, although it outperforms AudnnSynergy, MultiSyn, and DeepSynergy.

**Fig. 11:**
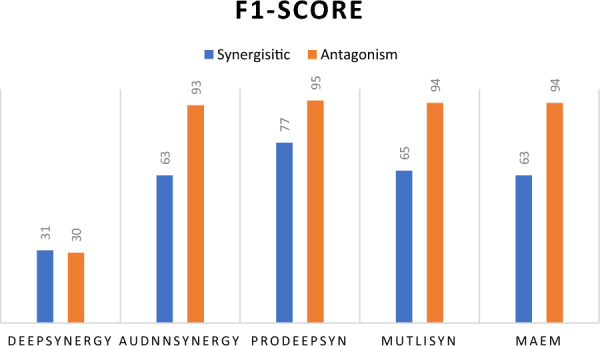
Comparative analysis of sixteen PR-AUC predictions values output from sixteen representation input to multi-task attention model and MAEM

Figure 11 depicts the F1-score for the synergistic and antagonistic classes. While MAEM is not the best model overall, it demonstrates strong performance in the antagonistic class with a high F1-score, indicating an effective balance between precision and recall. For the synergistic class, the F1-score is acceptable, matching that of AudnnSynergy and exceeding that of DeepSynergy.

## Conclusion

A novel approach (MAEM) employing a multi-task attention deep learning model with multi-view feature representation and an aggregate ensemble model has been introduced in this paper. The primary objective was the concurrent prediction of both the synergy class label and synergy score value for drug combinations. The model incorporated four distinct views for feature representation of each drug and cancer cell. The views included drug-target features, Mordred SMILES features, multi-view graph molecular features, and fingerprint features for drugs. For cell lines, the four views encompass gene expression profile features, copy number variation features, mutation features, and proteomics features. The model employs a multi-task learning approach where each drug view feature representation is paired with each cell view feature representation, resulting in sixteen output prediction values from the multi-task attention model for synergy score value and synergy class label based on the sixteen combined input representations. To consolidate these sixteen output predictions, an ensemble model has been applied, producing the final prediction value.

The effectiveness of MAEM has been assessed using the O'Neil benchmark cancer dataset, consisting of drug combinations and cancer cell line information. In contrast to preceding deep learning models employed for drug combination tasks, the MAEM model achieves noteworthy CC_P_ and demonstrates low MSE and RMSE when predicting synergy score values. Furthermore, in the prediction of synergy class labels, the MAEM model showcases elevated precision, ROC-AUC, PR-AUC, and Kappa values, outperforming previous models in these metrics.

To advance the understanding of drug combinations, it is essential to consider factors beyond synergy such as evaluating the potential side effects of drug combinations, as even highly synergistic combinations may present significant risks. Additionally, a thorough examination of the drug design can offer valuable insights into optimizing the combination process. By incorporating these broader considerations, it can make more informed and comprehensive decisions, ensuring that drug combinations are not only effective but also safe and well-regulated.

## Data Availability

The source code along with the O’Neil dataset datasets applied in this paper are accessible online at: [https://github.com/samar-monem/MAEM/tree/main].
